# Sinensol-C Isolated from *Spiranthes sinensis* Inhibits Adipogenesis in 3T3-L1 Cells through the Regulation of Adipogenic Transcription Factors and AMPK Activation

**DOI:** 10.3390/molecules25184204

**Published:** 2020-09-14

**Authors:** Pei-Hsin Shie, Chung-Ping Yang, Guan-Jhong Huang, Sheng-Yang Wang, Yueh-Hsiung Kuo

**Affiliations:** 1 Department of Biotechnology, School of Life Sciences, Longyan University, Longyan 364012, China; sps0220@gmail.com (P.-H.S.); cpyang218@gmail.com (C.-P.Y.); 2 Fujian Provincial Key Laboratory for the Prevention and Control of Animal Infectious Diseases and Biotechnology, Longyan University, Longyan 364012, China; 3 Key Laboratory of Preventive Veterinary Medicine and Biotechnology, Longyan University, Longyan 364012, China; 4 Department of Chinese Pharmaceutical Sciences and Chinese Medicine Resources, China Medical University, Taichung 404, Taiwan; gjhuang@mail.cmu.edu.tw; 5 Department of Forestry, National Chung Hsing University, Taichung 402, Taiwan; 6 Department of Biotechnology, Asia University, Taichung 413, Taiwan

**Keywords:** *Spiranthes sinensis*, sinensol-C, phenanthrene derivatives, adipogenesis, 3T3-L1 adipocytes

## Abstract

Obesity is an abnormal medical condition caused by accumulation of body fat that presents negative health impacts. Adipocyte hyperplasia, also known as adipogenesis, is one of the major manifestations of obesity. In the present study, we isolated six phenanthrene derivatives (compounds **1**–**6**) from the ethyl acetate fraction of *Spiranthes sinensis* and investigated their anti-adipogenic activity. We found that among the six phenanthrene derivatives, compound **6** (sinensol-C) exhibited strong inhibitory activity against intracellular lipid accumulation in 3T3-L1 adipocytes, with an IC_50_ value of 12.67 μM. Sinensol-C remarkably suppressed the accumulation of lipid droplets and adipogenesis, via down-regulation of adipogenic transcription factors, including peroxisome proliferator-activated receptor γ (PPARγ), CCAAT/enhancer binding protein α (C/EBPα), sterol regulatory element binding protein-1 (SREBP-1c), fatty acid synthase (FAS), and fatty acid binding protein 4 (FABP4), during adipocyte differentiation in 3T3-L1 cells. In addition, treatment with sinensol-C significantly increased the adenosine monophosphate-activated protein kinase (AMPK) activity in 3T3-L1 cells. Taken together, these data strongly suggest that sinensol-C regulates adiogenesis via down-regulation of adipogenic transcription factors and up-regulation of AMPK. Furthermore, this is the first study that demonstrates that sinensol-C has the capacity to modulate adipogenesis.

## 1. Introduction

*Spiranthes sinensis*, commonly known as the Chinese spiranthes, belongs to the family Orchidaceae and is widely distributed in Eastern Asia, including China, Japan, and Taiwan [1,2]. In traditional Chinese medicine, *S. sinensis* has been used to treat various human diseases such as sexual dysfunction, hemoptysis, epistaxis, headache, chronic dysentery, and meningitis [3]. In addition, modern scientific investigations have indicated that *S. sinensis* possesses various pharmacological activities, including being anti-HBV, anti-inflammatory, anti-oxidant, anti-bacterial, and anti-cancer [4,5,6,7,8]. Medicinal plants to prevent obesity have been widely investigated [9]. Many reports have indicated that bioactive compounds isolated and identified from plants are potentially useful to prevent or treat obesity [10,11,12]. Therefore, isolation of anti-adipogenic compounds from medicinal plants can provide therapeutic and preventive strategies for the development of new applications. *S. sinensis* contains various types of bioactive compounds, such as phenanthrenes, flavonoids, coumarins, and steroids [13,14,15,16]. Among them, phenanthrene is the major chemical component [16,17]. However, there is insufficient evidence to show the anti-adipogenesis effect of *S. sinensis.* Therefore, the first step of the present study is to isolate phenanthrene compounds and screen out potentially anti-adipogenic bioactive compounds from *S. sinensis.*

Obesity is defined based on the level of adipose tissue accumulation [18]. Obesity is a chronic metabolic disorder caused by an imbalance between energy intake and expenditure, and is recognized as a great public health problem [19]. In addition, obesity is associated with the development of many diseases, such as type-II diabetes mellitus, insulin resistance, hyperlipidemia, hypertension, cardiovascular diseases, and inflammation [20,21,22]. To combat obesity, many pharmacological treatments have been applied. However, these anti-obesity drugs involve diverse side effects such as insomnia, dry mouth, and dizziness [23]. For this reason, the development of anti-obesity drugs from natural products without, or with fewer, side effects is required.

Adipogenesis is a complex process that involves the differentiation of preadipocytes into adipocytes and lipid accumulation [24]. Adipocyte differentiation is mediated by the expression of various transcription factors and adipogenesis related genes, such as peroxisome proliferator-actuated receptors (PPARs), the CCAAT/enhancer binding protein (C/EBP) family, sterol regulatory element binding protein-1 (SREBP-1c), fatty acid synthase (FAS), fatty acid binding protein 4 (FABP4), and adiponectin [24,25]. Adenosine monophosphate-activated protein kinase (AMPK) is a regulator of cellular lipid metabolism and is one of the most well characterized anti-obesity targets. AMPK phosphorylation is able to suppress lipid accumulation via reducing the expression of several transcription factors, such as PPARγ, C/EBPα, and SREBP-1c, which are involved in lipid synthesis and associated processes [26,27,28]. Therefore, inhibition and modulation of these transcription factors and AMPK may be a key to regulating adipogenesis.

In the present study, we attempted to isolate anti-adipogenic constituents from *S. sinensis*. Six phenanthrene derivatives were isolated from the ethyl acetate fraction of *S. sinensis*, among them sinensol-C exhibited the best inhibitory effect against intracellular lipid accumulation in 3T3-L1 adipocytes. Our further investigation revealed that sinensol-C modulates adipogenic transcription factors through the activation of AMPK.

## 2. Results

### 2.1. Effect of the Compounds Isolated from S. Sinensis on Adipocyte Differentiation in 3T3-L1 Cells

**Figure 1:**
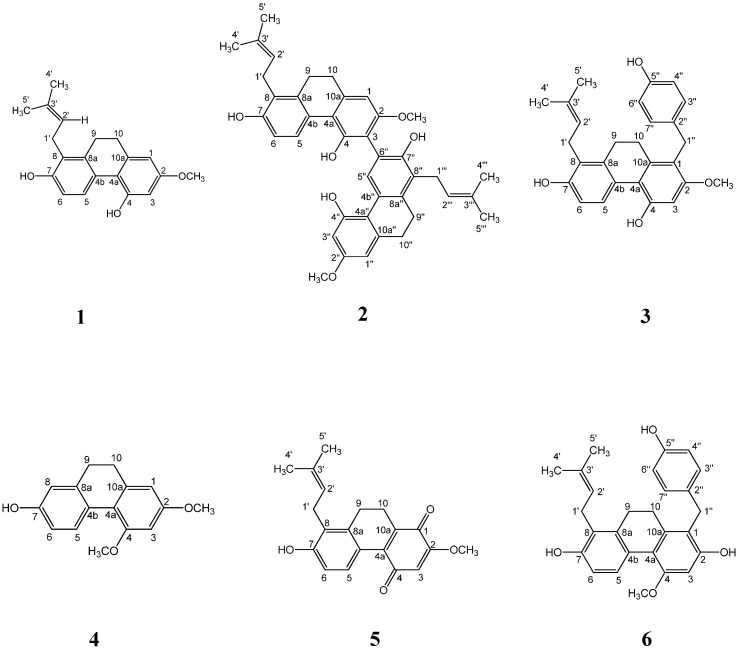
Chemical structures of compound **1**–**6**. spiranthol-A (**1**), spiranthesol (**2**), spirasineol-A (**3**), orchinol (**4**), spiranthoquinone (**5**) and sinensol-C (**6**).

**Figure 2:**
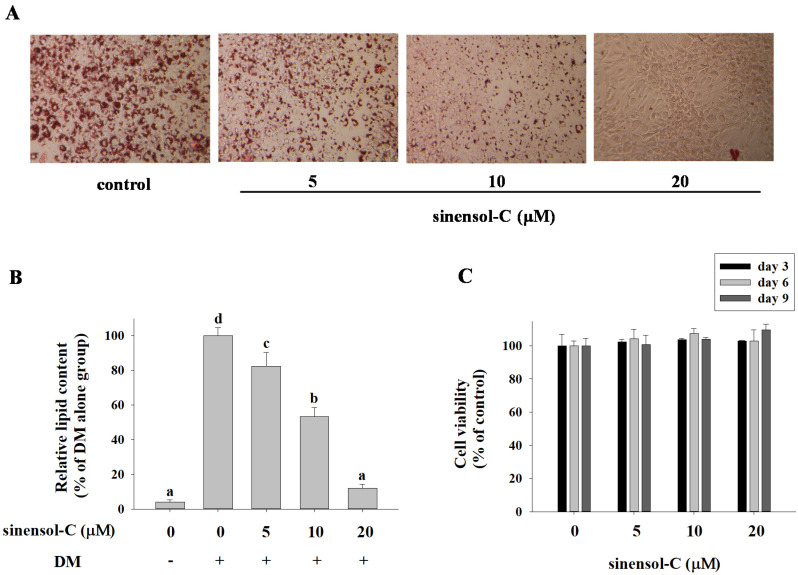
Effect of sinensol-C on intracellular lipid accumulation at day 9 in 3T3-L1 adipocytes. (**A**) Effects of sinensol-C on lipid droplet formation in 3T3-L1 adipocytes assessed by Oil Red O staining and visualized under light microscopy. (**B**) Relative lipid content of each sample determined by quantitative analysis of Oil Red O content. 3T3-L1 cells differentiated with differentiation media in the absence or presence of sinensol-C for 9 days. (**C**) Effects of sinensol-C on cell viability in 3T3-L1 cells. Cells were incubated with different concentrations (0, 5, 10, and 20 μM) of sinensol-C for 3, 6, and 9 days. DM: differentiation medium. Data are expressed as mean ± SD of three replicates. Different letters denote significant difference (*p* < 0.05) between the groups.

To examine the effect of the compounds (Figure 1) isolated from S. sinensis on the differentiation of preadipocytes into adipocytes, confluent 3T3-L1 preadipocytes were treated with various concentrations of compounds during the differentiation (days 0–9). After adipogenesis (day 9), the 3T3-L1 cells were stained with oil red O (ORO) solution to determine the accumulation of lipid droplets. Among the test compounds, spirasineol-A (**3**) and sinensol-C (**6**) exhibited inhibitory activities against lipid accumulation in adipocytes with IC_50_ values of 31.45 ± 2.48 and 12.67 ± 0.69 μM, respectively, compared to the activity of the positive control curcumin (IC_50_, 33.66 ± 0.95 μM). In particular, sinensol-C showed the most potent inhibitory activity against lipid accumulation in 3T3-L1 adipocytes (Table 1). As shown in Figure 2A, 3T3-L1 cells were treated with sinensol-C, which significantly inhibited the formation of intracellular lipid droplet accumulation, in a dose-dependent manner. Furthermore, 5, 10, and 20 μM sinensol-C significantly decreased the lipid content in 3T3-L1 cells by 82.4%, 53.4%, and 11.9%, respectively, compared with the content of fully differentiated adipocyte (Figure 2B). To confirm whether the anti-adipogenic effect of sinensol-C is due to its cytotoxicity, the cell viability was measured by MTT assay. The findings revealed that sinensol-C does not exhibit cytotoxicity in 3T3-L1 cells up a concentration of 20 μM (Figure 2C). Therefore, we have chosen the non-cytotoxic concentrations of sinensol-C for subsequent experiments.

### 2.2. Effect of Sinensol-C on Adipogenesis-Related Gene Expression during the Entire Differentiation Period

**Figure 3:**
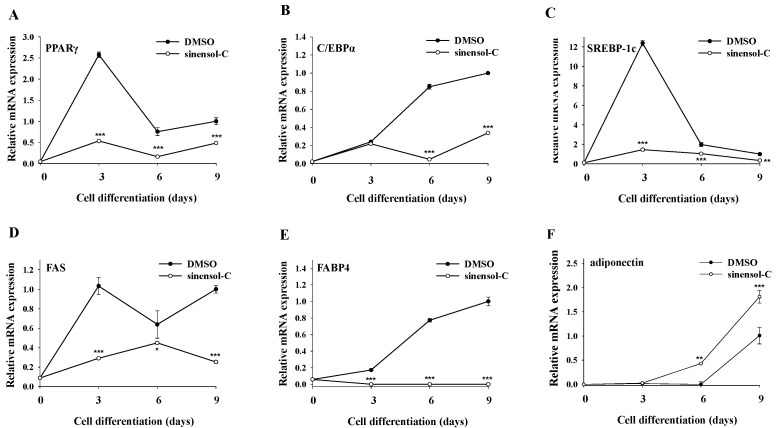
Effect of sinensol-C on the adipogenesis-related gene expression patterns during entire differentiation period (**A**–**F**). Two-day postconfluent 3T3-L1 preadipocytes (day 0) were treated with sinensol-C (20 μM) every 3 days for 9 days. Cells treated with 0.01% dimethyl sulfoxide (DMSO) were used as controls. At the indicated days after inducing differentiation, total RNA was isolated and mRNA levels of the indicated genes were measured by real-time quantitative RT-PCR. Results were expressed relative to untreated cells after normalization to 18S rRNA. Data are expressed as mean ± SD of three replicates. * *p* < 0.05, ** *p* < 0.01, and *** *p* < 0.001 were compared with control.

It was well demonstrated that PPARγ, C/EBPα, and SREBP-1c are critical transcription factors that regulate adipocyte differentiation and lipid production. Therefore, we examined whether sinensol-C treatment could modulate the expression of these marker genes in differentiated adipocytes. As shown in Figure 3A,C, treatment with sinensol-C significantly inhibited the mRNA expression levels of PPARγ and SREBP-1c at all differentiation periods (day 3, 6, and 9). Furthermore, sinensol-C also had a significant inhibitory effect on C/EBPα mRNA level, especially at day 6 and day 9 (Figure 3B). Next, we also evaluated the effect of sinensol-C on the expression of adipogenesis-related genes, such as FAS, FABP4, and adiponectin. As shown in Figure 3D,E, the mRNA levels of FAS and FABP4 were obviously suppressed by sinensol-C, when compared with the control group at all differentiation periods. It is notable that sinensol-C markedly enhanced the mRNA level of adiponectin, compared with the control at differentiation day 6 and 9 in 3T3-L1 adipocytes (Figure 3F).

### 2.3. Effect of Sinensol-C on the Expression of Adipogenesis-Related Protein in 3T3-L1 Adipocytes

**Figure 4:**
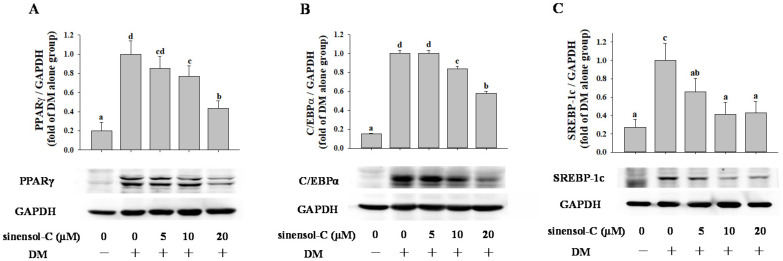
Effect of sinensol-C on the protein expression of differentiation related transcription factors (**A**–**C**). Confluent 3T3-L1 preadipocytes were differentiated into adipocytes in medium, either with or without different concentrations of sinensol-C for 9 days. DM: differentiation medium. The bands were normalized to an internal control (GAPDH), presented as the relative ratio. Data are expressed as mean ± SD of three replicates. Different letters denote significant difference (*p* < 0.05) between the groups.

**Figure 5:**
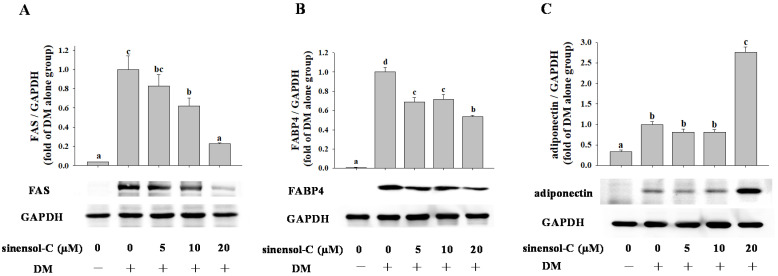
Effect of sinensol-C on the protein expression of fatty acid synthase (FAS) (**A**), FABP4 (**B**), and adiponectin (**C**). Confluent 3T3-L1 preadipocytes were differentiated into adipocytes in medium, either with or without different concentrations of sinensol-C for 9 days. DM: differentiation medium. The bands were normalized to an internal control (GAPDH), presented as the relative ratio. Data are expressed as mean ± SD of three replicates. Different letters denote significant difference (*p* < 0.05) between the groups.

According to previous results, sinensol-C not only down-regulated the mRNA level of transcription factors (PPARγ, C/EBPα, and SREBP-1c), it also interfered adipogenesis-specific genes (FAS, FABP4, and adiponectin) during the differentiation period of 3T3-L1 cells. Hence, we further examined whether sinensol-C modulated the protein levels in differentiated (day 9) 3T3-L1 cells by Western blot analysis. As shown in Figure 4, sinensol-C significantly and dose-dependently inhibited the protein level of PPARγ, C/EBPα, and SREBP-1c, suggesting that sinensol-C regulates adipogenesis by suppressing the expression of adipogenic transcription factors. In addition, treatment with sinensol-C significantly as well as dose-dependently down-regulated the protein levels of FAS and FABP4 in 3T3-L1 adipocytes (Figure 5A,B). Interestingly, compared with the control, the protein level of adiponectin was significantly increased by sinensol-C at a dose of 20 μM (Figure 5C), which is correlated with the increase of adiponectin mRNA level.

### 2.4. Effect of AMPK Activation on Adipocyte Differentiation in 3T3-L1 Cells

**Figure 6:**
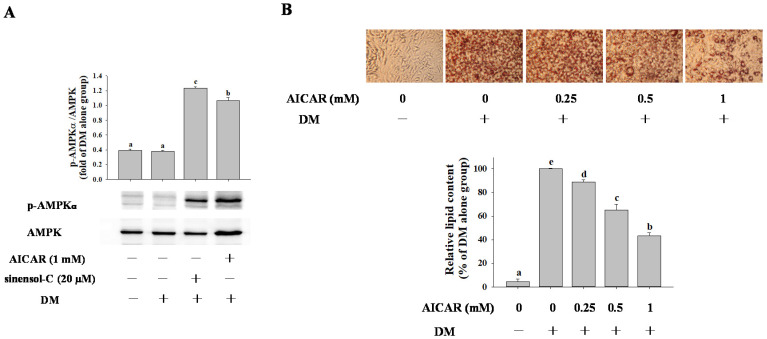
Effect of AICAR on (**A**) protein expression of p-AMPKα, and (**B**) intracellular lipid accumulation in 3T3-L1 adipocytes. 3T3-L1 cells differentiated with differentiation media in the absence or presence of AICAR for 9 days. The lipid droplet formation in 3T3-L1 adipocytes assessed by staining with Oil Red O and visualized under light microscopy. Relative lipid content of each sample determined by quantitative analysis of Oil Red O content. DM: differentiation medium. Data are expressed as mean ± SD of three replicates. Different letters denote significant difference (*p* < 0.05) between the groups.

To investigate the involvement of AMPK activation in modulating sinensol-C-mediated anti-adipogenesis effects, cells were treated with sinensol-C and AICAR (an activator of AMPK). We first confirmed whether the activation of AMPK with the sinensol-C was affected in 3T3-L1 cells. AMPK activity was measured by the amount of phosphorylation of AMPKα at threonine 172 residue (p-AMPKα) by immunoblotting at differentiation day 9. As shown in Figure 6A, sinensol-C (20 μM) and AICAR (1 mM) treatment significantly enhanced phosphorylation of AMPKα. In addition, we found that treatment with AICAR exhibited a dose-dependent inhibitory effect on lipid accumulation in differentiated adipocytes (Figure 6B).

**Figure 7:**
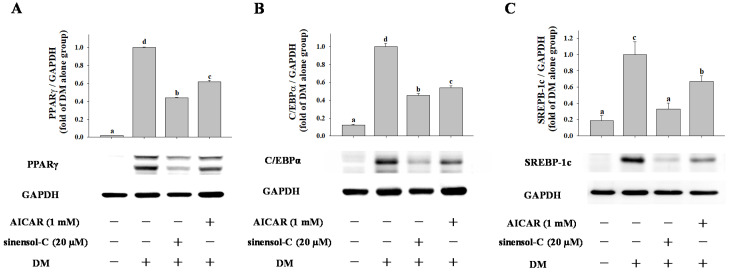
Effect of AICAR on the protein expression of key adipogenic transcription factors (**A**) PPARγ, (**B**) C/EBPα and (**C**) SREBP-1c in 3T3-L1 cells. Confluent 3T3-L1 preadipocytes were differentiated into adipocytes in medium either with or without sinensol-C (20 μM) or AICAR (1 mM) for 9 days. DM: differentiation medium. The bands were normalized to an internal control (GAPDH), presented as the relative ratio. Data are expressed as mean ± SD of three replicates. Different letters denote significant difference (*p* < 0.05) between the groups.

Next, we examined the effect of AICAR treatment on the protein expression of key transcriptional factors. As shown in Figure 7, compared with the control group, cells exposed to AICAR significantly blocked PPARγ, C/EBPα, and SREBP-1c protein expression by 0.44-fold, 0.46-fold, and 0.33-fold, respectively in 3T3-L1 cells. These results suggest that the anti-adipogenic effect of sinensol-C was associated with activation of AMPK in 3T3-L1 adipocyts.

## 3. Discussion

Obesity is a medical condition in which excessive fat accumulates in adipose tissue [29]. Therefore, suppression of lipid accumulation in adipocytes may be useful as an anti-obesity treatment. On that basis, we investigated natural product based therapeutic agents by evaluating their efficacy on intracellular lipid accumulation in adipocyte. Previous studies have reported that curcumin demonstrates excellent effects on anti-adipogenesis in 3T3-L1 cells [30,31,32]. Thus, we used curcumin as a positive control to suppress lipid accumulation in 3T3-L1 adipocytes, compared with the six phenanthrene derivatives from *S. sinensis* (Table 1). There is a growing interest in the search for an anti-adipogenic compound from medicinal plants. It is noteworthy that the anti-adipogenic activity of phenanthrene compounds from *S. sinensis* was far better than curcumin.

The phenanthrenes is abundant in the Orchidaceae family [33]. In the present study, we isolated six phenanthrene derivatives (compounds 1–6) from the whole plant of *S. sinensis*. A significant structure–activity relationship (SAR) among compound 1–6 was observed. The observed SAR implied that presence of 4-ethylphenol substituent on position 1 had an obvious influence on inhibition of lipid accumulation. Moreover, the hydroxy group at position 2 had stronger bioactivity than the methoxy group. However, the substitution on position 8 had no significant effect on bioactivity. Our data proved that compound 6 (sinensol-C), with 4-ethylphenol (at position 1) and hydroxy group (at position 2), exhibited more potent inhibitory activities than the other compounds.

In this study, sinensol-C (5~20 μM) suppressed lipid accumulation without exhibiting cytotoxicity during the differentiation of pre-adipocytes into adipocytes. Many studies have suggested that the differentiation of preadipocytes to mature adipocytes depends on a tightly regulated cascade of transcription factors, among which PPARγ, C/EBPα, and SREBP-1c are key regulators [25,34]. PPARγ plays a central role in adipogenesis and its absence blocks lipid droplet formation [35]. PPARγ activates the promoter of C/EBPα and vice versa, creating a positive feedback loop. In addition, PPARγ and C/EBPα induce multiple adipocyte-specific genes, including FABP4 and adiponectin [36,37]. SREBP-1c is involved in fatty acid synthesis and lipogenesis, with a series of activations to promote the expression of FAS [34,38]. In this study, Q-PCR and Western blot analysis revealed that sinensol-C down-regulated the expression of PPARγ, C/EBPα, and SREBP-1c, probably resulting in the reduced levels of FABP4 and FAS. PPARγ is a major regulator of adipocyte function and promotes the expression of adiponectin by binding to PPAR-responsive elements (PPRE), as heterodimers with retinoid X receptors (RXRs), and activates the target gene transcription [39,40,41]. However, our data showed that the expression of adiponectin was not reduced by the inhibition of PPARγ. A previous study indicated that liver receptor homolog-1 (LRH-1) also plays a significant role in the transcriptional activation of adiponectin gene via the LRH-responsive element (LRH-RE) in its promoter [42]. Therefore, we infer that sinensol-C increases the expression of adiponectin, maybe by promoting LHR-1, not PPARγ.

Adiponectin is one of the adipocyte-specific proteins secreted via adipose tissue, and has been shown to modulate lipid metabolism, glucose uptake, and energy expenditure [43,44]. There are epidemiological studies that indicate adiponectin levels are reduced in the plasma of patients with obesity, insulin resistance, or type II diabetes [45,46]. In addition, the studies have shown that adiponectin treatment enhances insulin-stimulated glucose phagocytosis by activating AMPK in primary adipocytes of rats, which has the potential to improve insulin resistance and type II diabetes [47,48]. Adiponectin also exhibits anti-hyperglycemic, anti-atherogenic, and anti-inflammatory properties, and could have important clinical benefits in terms of development of therapies for the prevention and/or the treatment of obesity, and obesity-related diseases [49]. Therefore, levels of adiponectin have been associated with obesity-related diseases. In this study, we found that sinensol-C plays a positive role in regulating adiponectin expression at the middle and terminal stage of adipogenesis in 3T3-L1 adipocytes. This result provided a positive feedback that sinensol-C may have the potential to treat obesity-related diseases.

AMPK is a major protein that regulates cellular energy homeostasis and regulates a number of biological pathways, including glucose levels and lipid metabolism [50,51]. Several studies have identified AMPK activation as a target for the treatment of obesity [52,53]. Accumulating evidence suggests that activation of the AMPK pathway suppressed PPARγ, C/EBPα, and SREBP-1c expression, and thus inhibits lipid accumulation during adipogenesis [54,55,56]. In addition, studies have related AMPK activation with the level of adiponectin [47,57,58,59]. Our results clearly indicated that sinensol-C not only inhibits the genes and proteins of adipogenic transcription factors (PPARγ, C/EBPα, and SREBP-1c), but also promotes expression of adiponectin. Based on this information, we speculated that sinensol-C has the potential to activate AMPK.

AICAR is a known activator of AMPK and has been used as an experimental agent to activate AMPK in vitro and in vivo [60,61]. It has been shown that administration of AICAR markedly blocks adipogenesis in adipocyte [59,62,63,64]. Therefore, we investigated whether sinensol-C inhibits adipogenesis, as well as AICAR, by activating AMPK. In this study, sinensol-C and AICAR significantly up-regulated phosphorylation of AMPK at threonine 172 residue. These results demonstrated that sinensol-C is an excellent AMPK activator, and thereby inhibits lipid accumulation via enhanced p-AMPKα expression, which negatively modulates levels of adipogenic transcription factors, and promotes levels of adiponectin in 3T3-L1 cells.

## 4. Materials and Methods

### 4.1. Chemicals and Reagents

Insulin, dexamethasone, 3-isobutyl-1-methylxanthine (IBMX), sodium pyruvate, N-2-hydroxyethylpiperazine-N’-2-ethanesulfonic acid (HEPES), Oil Red O, and 5-aminoimidazole-4-carboxamide ribonucleotide (AICAR) were purchased from Sigma-Aldrich Chemical Co., Inc. (St. Louis, MO, USA). Dubecco’s modified Eagle medium (DMEM), bovine serum (BS), fetal bovine serum (FBS), sodium pyruvate solution, and penicillin-streptomycin (PS) were purchased from Gibico (Invitrogen, Carlsbad, CA, USA). The polyclonal antibodies specific for GAPDH, PPAR-γ, C/EBPα, and p-AMPKα were purchased from Cell Signaling Technology, Inc. (Danvers, MA, USA). Anti-SREBP-1c antibody was obtained from Santa Cruz Biotechnology (Dallas, TX, USA). The rabbit polyclonal antibody for AMPKα was purchased from Millipore (Darmstadt, Germany). All chemicals and solvents of analytical grade were obtained from commercial sources.

### 4.2. Plant Material

Whole plants of *S. sinensis* were purchased from a local herbal medicine store in Taichung, Taiwan, and the specimen was verified by Dr. Shyh-Shyun Huang, professor of China Medical University. Voucher specimens (Specimen No. SS2011001) were maintained in the author’s laboratory. The purchased sample was air-dried and kept at 4 °C until use.

### 4.3. Isolation and Identification of Sinensol-C from S. Sinensis

The preparation of crude extracts of *S. sinensis* was previously described [5]. The resulting ethyl acetate fraction (254.2 g) was concentrated and purified by Silica gel (2000 g) column chromatography using a gradient of increasing polarity with *n*-hexane/ethyl acetate (100:0-0:100) as mobile phase, and separated into 15 subfractions (E1–E15) on the basis of TLC analysis. Fraction E4 (9.58 g) was fractioned after repeated chromatography over silica gel (*n*-hexane/acetone, *v*/*v* 9:1/2:1) to afford Fr. 4-1–4-6. Fr. 4-4 (1.44 g) was chromatographed on semi-preparative HPLC (*n*-hexane/acetone, *v*/*v* 3:1) to afford Orchinol (101.2 mg). Fraction E5 (8.74 g) was chromatographed on semi-preparative HPLC (*n*-hexane/acetone, *v*/*v* 7:3) to afford spiranthol-A (70.9 mg), spiranthesol (19.7 mg), spirasineol-A (12.9 mg). Fraction E6 (6.03 g) was eluted from silica gel using *n*-hexane/acetone gradient (*v*/*v* 95:5~100:0) as a mobile phase to afford seven subfractions (Fr. 6-1–6-7). Fr. 6-1 (11.12 g) were further isolated by semi-preparative HPLC with *n*-hexane/ acetone (*v*/*v* 2:1) to obtained spiranthoquinone (17.5 mg). Fraction 6-2 was purified by Sephadex LH20 column with dichloromethane/methanol (*v*/*v* 1:1) to obtained sinensol-C (3.02 g). The known compounds were identified as orchinol (**1**) [16,65], spiranthol-A (**2**) [16], spiranthesol (**3**) [13], spirasineol-A (**4**) [16], spiranthoquinone (**5**) [13], and sinensol-C (**6**) [4] by comparison of their physical and reported spectroscopic data. The purity of all compounds obtained was higher than 99% based on the results of HPLC and ^1^H NMR analyzed.

### 4.4. Cell Culture and Adipocyte Differentiation

**Figure 8:**
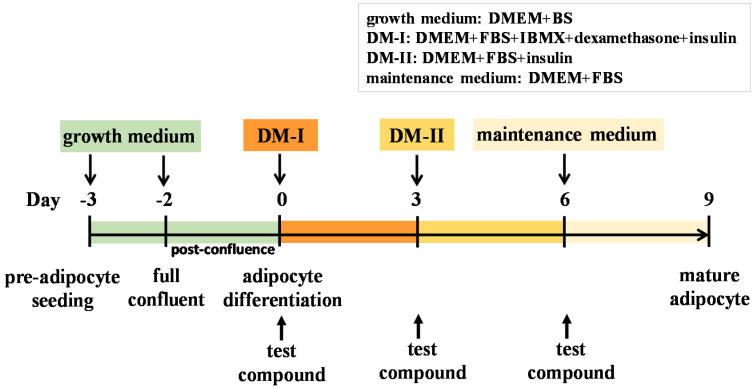
Scheme of 3T3-L1 pre-adipocyte differentiation and the treatment of test compounds.

The murine pre-adipocyte cell line (3T3-L1) was purchased from the Bioresource Collection and Research Center (BCRC, Hsinchu, Taiwan). Cells were cultured in 10 cm cell culture dishes containing DMEM supplemented with 10% BS, 1 mM sodium pyruvate, and 10 mM HEPES at 37 °C in a humidified incubator containing 5% CO_2_. The cells were differentiated into adipocytes according to the previously described protocol with minor modification [66]. 3T3-L1 pre-adipocytes were maintained post-confluence in growth medium for 2 days. The medium was replaced by DM-I (DMEM containing 10% FBS, 10 mM HEPES, 1 mM sodium pyruvate, 0.5 mM IBMX, 1 μM dexamethasone, and 10 μg/mL insulin), and this was defined as day 0 of differentiation induction. Cells were cultured in DM-I for 3 days (from day 0 to day 3). The DM-I was then replaced with DM-II (DMEM containing 10% FBS, 10 mM HEPES, 1 mM sodium pyruvate, and 10 μg/mL insulin) for another 3 days (day 3 to day 6). Cultures were incubated for 3 days, after the DM-II was replaced with maintenance medium (DMEM containing 10% FBS, 10 mM HEPES, 1 mM sodium pyruvate) after another 3 days and the cells were cultured up to day 9. The timescale of 3T3-L1 preadipocyte differentiation is shown in Figure 8. Test compounds were dissolved in dimethyl sulfoxide (DMSO) to a final concentration of 0.1% in media. The compounds were added to the medium on day 0, and added at the time of every medium change during the 9 days of incubation.

### 4.5. Cell Viability

Cell viability was determined by methylthiazolyldiphenyl-tetrazolium bromide (MTT) colorimetric assay. Briefly, 3T3-L1 pre-adipocytes at a density of 5 × 10^3^ cells/well were seeded in 96-well plates to full confluence. Two days after confluence, different concentrations of sinensol-C (5, 10 and 20 μM) were added to the DM-I (at day 0), DM-II (at day 3), and maintenance medium (at day 6), respectively, according to Figure 8. Sinensol-C was added to the medium at different concentrations (5, 10, and 20 μM) and then incubated for 3, 6, and 9 days. After treatment, the cells were incubated with 100 μL of MTT solution (0.5 mg/mL) for 4 h at 37 °C. The culture medium was discarded, and the cells were dissolved in DMSO. The optical density (OD) at 570 nm was measured using a micro-plate reader (µQuant, Bio-Tek Instruments, Inc., Winooski, VT, USA).

### 4.6. Oil Red O Staining

Lipid droplets in cells were stained with oil red O (ORO). The 3T3-L1 adipocytes (1 × 10^4^ cells/well) were seeded in 6-well plates. After differentiation, cells were washed twice with phosphate-buffered saline (PBS) and fixed with 10% formalin in PBS for 1 h at room temperature. After fixation, cells were washed twice with PBS and stained with a filtered ORO solution (6 parts saturated 0.3% ORO in isopropanol and 4 parts distilled water) for 10 min at room temperature. Subsequently, cells were washed thrice with PBS then visualized using a microscope (Olympus, Tokyo, Japan). To quantify the intracellular lipids, the cells were eluted with 100% isopropanol and the lipid accumulation was quantified by measuring the OD 510 nm using a microplate reader. The results were confirmed by three independent experiments.

### 4.7. Western Blot Analysis

After treatment, cells were lysed with mammalian protein extraction reagent (Thermo Scientific) containing protease inhibitor. Cell lysates were centrifuged (at 15,000× *g* for 15 min at 4 °C and the supernatants were used for Western blot analyses. Equal amounts of total protein (30 μg/lane) were separated by 10% SDS-PAGE and transferred onto poly-vinylidene difluoride (PVDF) membrane (Millipore, Bedford, MA, USA) at 110 V for 90 min. Membranes were blocked for 1 h at room temperature with 5% non-fat dry milk in PBS-Tween 20 (PBST, 0.1%), and incubated with the primary antibodies of proteins of interest. The membranes were incubated with the corresponding anti-rabbit or anti-mouse antibodies’ secondary antibodies, which were conjugated with horseradish peroxidase for 2 h at room temperature. The immuno blot bands were visualized by using an enhanced chemiluminescence substrate (Millipore) and were scanned by a VL Chemi-Smart 3000 image system (Viogene Biotek, Sunnyvale, CA, USA).

### 4.8. RNA Extraction and Q-PCR Analysis

Total RNA from 3T3-L1 cells were isolated with Trizol Reagent (Invitrogen) according to the manufacturer’s instructions. First strand cDNA synthesis from 5 μg of total RNA was performed using SuperScript™ III reverse transcriptase (Invitrogen) primed by oligo(dT)12-18 primer. PCR was performed using the indicated gene-specific primers given in Table 2. PCR products were measured with a StepOnePlus Real-time PCR System (Applied Biosystem, Foster City, CA, USA) and the relative gene expression was calculated based on the comparative CT values using a StepOne Software v2.0 (Applied Biosystems). The real-time PCR mixture, with a final volume of 20 μL, consisted of Power SYBR Green PCR Master Mix (Applied Biosystems), 1 μM of a forward primer, 1 μM of a reverse primer, and 0.5 μg of a cDNA sample. The thermal cycling conditions were: 10 min at 95 °C, 40 cycles of 15 s at 95 °C, and 60 s at 60 °C. Melt curve analyses were performed for all genes, and the specificity as well as integrity of the PCR products was confirmed by the presence of a single peak. The expression of 18S mRNA was used as an endogenous control.

### 4.9. Data Processing and Statistical Analysis

All data were expressed as mean ± standard deviation (SD) of three replicates. Data were analyzed using SPSS version 20.0 statistical software (IBM Corp., Armonk, NY) via one-way ANOVA followed by *Scheffé* multiple range tests. The criterion for statistical significance was set at *p* < 0.05.

## 5. Conclusions

In the present study, we examined the anti-obesity effects of sinensol-C on adipocyte differentiation and the associated mechanisms in 3T3-L1 cells. We found that sinensol-C significantly attenuated lipid accumulation and adipocyte differentiation of 3T3-L1 cells in a dose-dependent manner. Treatment with sinensol-C down-regulated the expression of the key transcriptional regulator (PPARγ, C/EBPα, and SREBP-1c) and subsequently reduced the levels of FABP4 and FAS in 3T3-L1 adipocytes. Sinensol-C also increased expression of adiponectin in adipocytes cells. Moreover, we observed that sinensol-C treatment could increase AMPK phosphorylation, which subsequently inhibits the protein expression of transcriptional regulators in adipocyte cells. These results suggest that sinensol-C could be a promising natural anti-adipogenic compound for the management of obesity. These findings may provide a detailed description of mechanisms underlying the anti-obesity effects of sinensol-C.

## Figures and Tables

**Table 1:** Inhibitory effect of phenanthrene derivatives from *Spiranthes sinensis* against intracellular lipid accumulation in 3T3-L1 adipocytes.

Compound	IC_50_ (μM) ^a^
spiranthol-A (**1**)	>60
spiranthesol (**2**)	38.93 ± 0.83
spirasineol-A (**3**)	31.45 ± 2.48
orchinol (**4**)	>60
spiranthoquinone (**5**)	>60
sinensol-C (**6**)	12.67 ± 0.69
curcumin ^b^	33.66 ± 0.95

^a^ IC_50_ means the 50% inhibitory concentration (μM) on intracellular lipid accumulation at day 9 in 3T3-L1 adipocytes. The IC_50_ values were calculated from the slope of the dose-response curves. Data are expressed as mean ± SD of three replicates. ^b^ Curcumin was used as a positive drug control.

**Table 2:** Sequences of primers used for quantitative RT-PCR.

Genes	Sequence
PPAR-γ	F: 5′-CAA GAA TAC CAA AGT GCG ATC AA-3′
	R: 5′-GAG CTG GGT CTT TTC AGA ATA ATA AG-3′
C/EBPα	F: 5′-AGC AAC GAG TAC CGG GTA CG-3′
	R: 5′-TGT TTG GCT TTA TCT CGG CTC-3′
SERBP-1c	F: 5′-GAT CAA AGA GGA GCC AGT GC-3′
	R: 5′-TAG ATG GTG GCT GCT GAG TG-3′
FAS	F: 5’-CCC TTG ATG AAG AGG GAT CA-3’
	R: 5′-ACT CCA CAG GTG GGG AAC AAG-3′
FABP4	F: 5′-AGT GAA AAC TTC GAT GAT TAC ATG AA-3′
	R: 5′-GCC TGC CAC TTT CCT TGT G-3′
adiponectin	F: 5′-TCC TGG AGA GAA GGG AGA GAA AG-3′
	R: 5′-TCA GCT CCT GTC ATT CCA ACA T-3′
18S rRNA	F: 5′-CGC CGC TAG AGG TGA AAT TCT-3′
	R:5′-CAT TCT TGG CAA ATG CTT TCG-3′

F: forward; R: reverse.
